# Supplementary material for: In-situ spectroscopic observation of dynamic-coupling oxygen on atomically dispersed iridium electrocatalyst for acidic water oxidation
Source: Nat Commun. 2021 Oct 21;12:6118. doi: 10.1038/s41467-021-26416-3 (PMC8531441; doi:10.1038/s41467-021-26416-3)
Supplement: Supplementary file 1 — Supplementary_Information [file 41467_2021_26416_MOESM1_ESM.docx]

**Supplementary Information**

***In-situ* spectroscopic observation of dynamic-coupling oxygen on atomically dispersed iridium electrocatalyst for acidic water oxidation**

Hui Su,^1,†^ Wanlin Zhou,^1,†^ Wu Zhou,^2^ Yuanli Li,^1^ Lirong Zheng,^3^ Hui Zhang,^1^ Meihuan Liu,^1^ Xiuxiu Zhang,^1^ Xuan Sun,^1^ Yanzhi Xu,^1^ Fengchun Hu,^1^ Jing Zhang,^3^ Tiandou Hu,^3^ Qinghua Liu,^1,^* and Shiqiang Wei^1,^*

^1^*National Synchrotron Radiation Laboratory, University of Science and Technology of China, Hefei 230029, Anhui, P. R. China*

^2^*School of Chemistry and Chemical Engineering, Key Laboratory for Green Processing of Chemical Engineering of Xinjiang Bingtuan, Shihezi University, Shihezi 832003, China*

^3^*Beijing Synchrotron Radiation Facility, Institute of High Energy Physics, Chinese Academy of Sciences, Beijing 100049, China*

^†^*These authors contributed equally: Hui Su, Wanlin Zhou.*

*E-mail: qhliu@ustc.edu.cn; sqwei@ustc.edu.cn

**Contents:**

Supplementary Figs. 1-29;

Supplementary Tables 1-6;

Supplementary references 1-16.

**Supplementary Fig. 1.** SEM images of PAni-CP (a) before and (b) after carbonization. SEM images of NH_2_- PAni-CP (a) before and (b) after carbonization.

**Supplementary Fig. 2.** SEM images of Ir-NC (a) (b) and AD-HN-Ir electrocatalysts (c) (d).

**Supplementary Fig. 3.** SEM images of Ir-NP/NC

**Supplementary Fig. 4.** TEM images of Ir-NC (a) and AD-HN-Ir electrocatalyst (b).

**Supplementary Fig. 5.** (a) Atomic-resolution HAADF-STEM image of AD-HN-Ir electrocatalyst, (b) intensity profile along the line 1 in (a) and (c) intensity profile along the line 2 and 3 in (a), indicating that Ir atoms exists exclusively uniform distribution.

**Supplementary Fig. 6.** (a) XRD patterns for AD-HN-Ir, NC and Ir-NC electrocatalysts.

**Supplementary Fig. 7.** (a) XPS survey spectra of AD-HN-Ir electrocatalyst. The high-resolution spectra of C 1s (b).

**Supplementary Fig. 8.** (a) Ir *L*_3_-edge XANES spectra, (b) *k*^2^χ(*k*) oscillations of Ir *L*_3_-edge XANES oscillation functions and (c) Fourier transforms (FTs) of the Ir *L_3_*-edge EXAFS oscillations of AD-HN-Ir electrocatalyst, Ir-NP/NC, Ir foil, IrO_2_, and the fitting curves of *k*^2^-weighted EXAFS spectra of AD-HN-Ir electrocatalyst.

**Supplementary Fig. 9.** (a) OER polarization curves of AD-HN-Ir electrocatalyst, PAni-Ir-800˚C and IrO_2_ in O_2_-saturated 0.5 M H_2_SO_4_. (b) The corresponding overpotentials at current density of 10, 50 and 100 mA cm^-2^.

**Supplementary Fig. 10.** (a) Linear sweep voltammetry (LSV) curves for Ir-based catalysts annealed at 700, 800 and 900 ˚C. SEM image of Ir-based catalysts annealed at (b) 700 ˚C, (c) 800 ˚C and (d) 900 ˚C.

**Supplementary Fig. 11.** Linear sweep voltammetry (LSV) curves of AD-HN-Ir electrocatalyst under 25, 50 and 80 ˚C in 0.5 M H_2_SO_4_.

**Supplementary Fig. 12.** Double-layer capacitance measurements. (a), (c) CVs were conducted in a non-Faradaic region of voltammogram at the following scan rate: 0.01, 0.05, 0.1, 0.5, 1 V s^-1^. (b), (d) The difference in charging currents variation at an underpotential plotted against scan rate for estimation of double-layer capacitance (C_dl_).

**Supplementary Fig. 13.** Double-layer capacitance measurements. (a) CVs were conducted in a non-Faradaic region of voltammogram at the following scan rate: 0.01, 0.05, 0.1, 0.5, 1 V s^-1^, 0.07, 0.08, 0.09 and 0.10 V s^-1^. (b) The difference in charging currents variation at an underpotential plotted against scan rate for estimation of double-layer capacitance (C_dl_).

**Supplementary Fig. 14.** (a) OER polarization curves of AD-HN-Ir, Ir-NC and IrO_2_ in O_2_-saturated 0.5 M H_2_SO_4_ based on the ECSA.

**Supplementary Fig. 15.** Linear sweep voltammograms (LSVs) of AD-HN-Ir (a) and IrO_2_ (b) at different temperatures.

**Supplementary Fig. 16.** Tafel curves of AD-HN-Ir electrocatalyst (a) and IrO_2_ (b) at different temperatures. The exchange current density j_0_ can be calculated by Tafel curves, according to the Arrhenius equation (log j_0_=log(FK_C_) −ΔG_0_/(2.303RT), where R is the gas constant, ΔG_0_ is the apparent activation energy, F is the Faraday constant). This result infers that AD-HN-Ir electrocatalyst show higher OER activity with the lower activation energy of reaction.

**Supplementary Fig. 17**. (a) (b) Gas chromatography survey during OER tests for AD-HN-Ir electrocatalyst. (c) Generated O_2_ volumes over time versus theoretical quantities assuming ~97.5% Faradaic efficiency with the inset showing the Faradaic efficiencies of AD-HN-Ir electrocatalyst for O_2_ production at different bias potentials (d).

**Supplementary Fig. 18.** OER measurements using the two-electrode-cell in 0.5 M H_2_SO_4_ solution, where Pt/C and AD-HN-Ir electrocatalyst were employed as the cathode and anode for full-water splitting.

**Supplementary Fig. 19.** OER polarization curves of AD-HN-Ir electrocatalyst before and after 3000 electrochemical test cycles.

**Supplementary Fig. 20.** (a) Ir 4f XPS spectra, (b) and Ir L_3_ EXAFS, (c) and (d) SEM images for AD-HN-Ir electrocatalyst before and after electrochemical measurements.

**Supplementary Fig. 21.** OER stability for AD-HN-Ir electrocatalyst at applied potential of 1.53 V (a) and 1.75 V (c), and SEM image (b), (d) of AD-HN-Ir electrocatalyst after reaction.

 **Supplementary Fig. 22**. (a) Dissolved content of metal Ir, (b) the calculated constant S-number under 1.53 and 1.75 V vs. RHE. Error bars were obtained by standard deviation of at least three independent measurements. (c) Calculation of lifetime for AD-HN-Ir electrocatalyst.

**Supplementary Fig. 23.** (a) Membrane electrode assembly. (b) The cell voltage of the PEM electrolyser system held at 500 and 1000 mA cm^-2^. (c) PEM electrolyser cell and (d) a PEM electrolyser system.

**Supplementary Fig. 24.** The equivalent circuits of OER. (a) 0.95-1.10 V for AD-HN-Ir electrocatalyst and 0.95-1.15 V for Ir-NC. (b) 1.15-1.40 V for AD-HN-Ir electrocatalyst and 1.20-1.45 V for Ir-NC.

**Supplementary Fig. 25.** In situ SRIR measurements (a) with smoothing and (b) without smoothing in the range of 1300–600 cm^-1^ under various potentials for AD-HN-Ir electrocatalyst during the OER process.

**Supplementary Fig. 26.** The *in-situ* SRIR signal. In the range 1300-600 cm^-1^ under various potentials for Ir-NC during electrocatalytic OER process.

**Supplementary Fig. 27.** *In situ* SRIR measurements in the range of 1300–600 cm^-1^ under 1.45V and after reaction (A. R.) conditions for AD-HN-Ir electrocatalyst.

**Supplementary Fig. 28.** (a) *k*^2^χ(*k*) curves of Ir *L*-edge EXAFS oscillation functions. Corresponding *k*^2^-weighted FT of Ir *L_3_*-edge EXAFS oscillation functions (b) under different R_bkg_ values of 0.9, 1.0 and 1.1 Å for background removal and (c) at four different *k* ranges for AD-HN-Ir electrocatalyst. (d) FT spectrum of Ir *L_3_*-edge EXAFS oscillation functions for AD-HN-Ir, Ir foil and IrO_2_. (e) The fitting curve of *k*^2^-weighted EXAFS spectrum and (f) the Re(*k*^2^χ(*k*)) oscillation curve for AD-HN-Ir electrocatalyst under 1.45 V.

**Supplementary Fig. 29.** The fitting curves of *k*^2^-weighted EXAFS spectra and the *Re*(*k*^2^χ(*k*)) oscillation curves for AD-HN-Ir electrocatalyst (a), (b) under 1.25 V, (c), (d) at 1.35 V and (e), (f) at 1.45 V conditions.

**Supplementary Table 1.** Structural parameters for AD-HN-Ir electrocatalyst extracted from quantitative EXAFS curve-fitting using the ARTEMIS module of IFEFFIT.

N, coordination number; R, bond length; σ^2^ Debye-Waller factor; ΔE_0_ inner potential shift.

**Supplementary Table 2.** Comparison of OER activity of AD-HN-Ir electrocatalyst with other recently reported catalysts in acid solution. ^[1-16]^

**Supplementary Table 3.** The EIS fitting data of AD-HN-Ir electrocatalyst.

**Supplementary Table 4.** The EIS fitting data of Ir-NC.

**Supplementary Table 5.** Structural parameters for the AD-HN-Ir electrocatalyst extracted from quantitative EXAFS curve-fitting by considering the first-shell of Ir-N/O and Ir-Ir coordination.

N, coordination number; R, bond length; σ^2^ Debye-Waller factor; ΔE_0_ inner potential shift.

**Supplementary Table 6.** Structural parameters for AD-HN-Ir electrocatalyst under different potentials extracted from quantitative EXAFS curve-fitting using the ARTEMIS module of IFEFFIT.

N, coordination number; R, bond length; σ^2^ Debye-Waller factor; ΔE_0_ inner potential shift.

**Supplementary References**

1 Oh, H. *et al*. Electrochemical catalyst–support effects and their stabilizing role for IrO_x_ nanoparticle catalysts during the oxygen evolution reaction. *J. Am. Chem. Soc.* **138**, 12552-12563 (2016).

2 Park, J., Sa, Y., Baik, H., Kwon, T., Joo, S. & Lee, K. Iridium-based multimetallic nanoframe@nanoframe structure: An efficient and robust electrocatalyst toward oxygen evolution reaction. *ACS nano* **11**, 5500-5509 (2017).

3. Yang, L. *et al*. Efficient oxygen evolution electrocatalysis in acid by a perovskite with face-sharing IrO6 octahedral dimers. *Nat. Commun.* **9**, 5236 (2018).

4. Kim, J., Shih, P., Qin, Y., A-Bardan, Z., Sun, C. & Yang, H. A porous pyrochlore Y_2_[Ru_1.6_Y_0.4_]O_7–δ_ electrocatalyst for enhanced performance towards the oxygen evolution reaction in acidic media. *Angew. Chem. Int. Ed.* **130**, 14073-14077 (2018).

5. Laha, S. *et al*. Oxygen evolution catalysis: Ruthenium oxide nanosheets for enhanced oxygen evolution catalysis in acidic medium. *Adv. Energy. Mater.* **9**, 1803795 (2019).

6. Cao, L. *et al*. Dynamic oxygen adsorption on single-atomic Ruthenium catalyst with high performance for acidic oxygen evolution reaction. *Nat. Commun.* **10**, 4849 (2019).

7. Yao, Y. *et al*. Engineering the electronic structure of single atom Ru sites via compressive strain boosts acidic water oxidation electrocatalysis. *Nat. Catal.* **2***, 304-313* (2019).

8. Lin, Y. *et al*. Chromium-ruthenium oxide solid solution electrocatalyst for highly efficient oxygen evolution reaction in acidic media. *Nat. Commun.* **10**, 162 (2019).

9. Nong, H. *et al*. IrOx core-shell nanocatalysts for cost-and energy-efficient electrochemical water splitting. *Chem. Sci.,* **5**, 2955-2963 (2014).

10. Grimaud, A. *et al*. Activation of surface oxygen sites on an iridium-based model catalyst for the oxygen evolution reaction. *Nat. Energy* **2**, 16189 (2016).

11. Diaz-Morales, O. *et al*. Iridium-based double perovskites for efficient water oxidation in acid media. *Nat. Commun.* **7**, 12363 (2016).

12. Seitz, L. *et al*. A highly active and stable IrOx/SrIrO3 catalyst for the oxygen evolution reaction. *Science* **353**, 6303 (2016).

13. Gao, J. *et al*. Breaking long-range order in iridium oxide by alkali ion for efficient water oxidation. *J. Am. Chem. Soc.* **141**, 3014(2019).

14. Zhao, Y. *et al*. 3D nanoporous iridium-based alloy microwires for efficient oxygen evolution in acidic media. *Nano Energy* **59**, 146-153 (2019).

15. Faustini, M. *et al*. Hierarchically Structured Ultraporous Iridium-Based Materials: A Novel Catalyst Architecture for Proton Exchange Membrane Water Electrolyzers. *Adv. Energy Mater.* **9**, 1802136 (2019).

16. Su, J. *et al*. Assembling ultrasmall copper-doped ruthenium oxide nanocrystals into hollow porous polyhedra: Highly robust electrocatalysts for oxygen evolution in acidic media. *Adv. Mater.* **30**, 1801351(2018).
